# The Mediating Effect of Depression on the Relation Between Interpersonal Needs and Suicidal Ideation Among Chinese Transgender Women

**DOI:** 10.3389/fpubh.2021.764198

**Published:** 2022-01-20

**Authors:** Ruijie Chang, Chengbo Zeng, Shan Qiao, Huwen Wang, Chen Xu, Xiaoyue Yu, Tiecheng Ma, Ying Wang, Xiaoming Li, Yong Cai

**Affiliations:** ^1^School of Public Health, School of Medicine, Shanghai Jiao Tong University, Shanghai, China; ^2^Department of Health Promotion, Education and Behavior, Arnold School of Public Health, University of South Carolina, Columbia, SC, United States; ^3^South Carolina SmartState Center for Healthcare Quality, Arnold School of Public Health, University of South Carolina, Columbia, SC, United States; ^4^Big Data Health Science Center, University of South Carolina, Columbia, SC, United States; ^5^China Love Aid, Shenyang, China

**Keywords:** transgender women, suicidal ideation, depression, mediating effect, interpersonal theory of suicide

## Abstract

**Background:**

Transgender women are at high risk of depression and suicidal ideation. The interpersonal theory of suicide proposes that suicidal ideation could be a consequence of high interpersonal needs (thwarted belongingness and perceived burdensomeness). The current study tests this theory and investigates whether depression could mediate the relationship between interpersonal needs and suicidal ideation among transgender women in Shenyang, China.

**Methods:**

A total of 198 transgender women were recruited by snowball sampling. A cross-sectional study was conducted through a structured questionnaire. Suicidal ideation, depression, and interpersonal needs were assessed. Path analysis was used to carry out the research goals and the mediating effect of depression was tested.

**Results:**

There were nearly 37% of the participants reported lifetime suicidal ideation. Suicidal ideation was positively correlated with thwarted belongingness (*t* = −5.53, *p* < 0.01) and perceived burdensomeness (*t* = −5.02, *p* < 0.01). The direct effect from thwarted belongingness to suicidal ideation *via* depression was statistically significant (Std. β = 0.232, *p* < 0.01). Depression could also mediate the indirect path from perceived burdensomeness to suicidal ideation through depression (Std. β = 0.222, *p* < 0.01) although the direct path between them was not significant (Std. β = 0.046, *p* = 0.693).

**Conclusions:**

Depression fully mediated the relationship between perceived burdensomeness and suicidal ideation, and partially mediate the relationship between thwarted belongingness and suicidal ideation. To reduce the risk of suicidal ideation among transgender women, interventions targeting thwarted belongingness, perceived burdensomeness, and depression are needed.

## Introduction

Transgender women are individuals who were assigned male at birth but identify themselves as women (1). It is estimated that transgender women accounted for 0.5–1.3% of the world's population (2). In China, there are about 7,000,000 transgender women, accounting for 7.28–18.92% of the overall world transgender women population. Transgender women are vulnerable to mental health problems, such as suicidal ideation, depression, anxiety, and suicide (3–6). A large representative study among 1,309 transgender population across 32 provinces and municipalities in China found that transgender women had a higher prevalence of suicidal ideation (56.4%) (7) than the general population (3.1%) (8). Based on the suicide theory, suicidal ideation has a close association with suicide attempt, suicide-related injury, and deaths (9). Studies investigating the facilitators of suicidal ideation could inform effective interventions to reduce the suicide attempt, injuries, and deaths among transgender women (10).

Prior research had conceptualized the mechanisms of suicidal ideation among various populations. Joiner's interpersonal theory of suicide assumes that people with the most determined suicidal ideation often experience interpersonal needs (high thwarted belongingness and perceived burdensomeness) (11). Thwarted belongingness refers to the belief that someone has no social connectedness, which could result in a desire for death (12). Perceived burdensomeness represents the feeling that one is a burden on significant others or the society, including but not limited to family members (12). As a highly vulnerable and marginalized gender minority, transgender women in China (13) often suffer stigma and discrimination in their daily life, take high risks of losing jobs and better education opportunities, and face barriers accessing healthcare services. Thus, they are more likely to experience depression and suicide and more attention should be paid to the social support and mental health of transgender women (14, 15). Social isolation induced from rejection from people around, and life situations, such as homelessness and unemployment, which are of high incidence among transgender women, may result in feelings of both thwarted belongingness and perceived burdensomeness (16). The interpersonal theory of suicide could be applied to transgender women and provide an in-depth understanding of the high prevalence of suicide in this unique population. Several studies have also tested the interpersonal theory of suicide among transgender people (16, 17). Testa demonstrated in an analysis of a convenience sample of 816 transgender and gender non-conforming (TGNC) adults that the interpersonal theory of suicide conferred risk for suicidal ideation among TGNC individuals. However, the processes or mechanisms are understudied, few studies have explored whether psychological disorders mediate the pathway from interpersonal needs to suicidal ideation.

Depression is closely associated with suicidal ideation (18), with about a half to two-thirds with suicidal ideation having depression in transgender people (19, 20). A study among transgender women in Canada found that more than 60.0% of them suffered from depression, which was much higher than the cisgender populations (21). A meta-analysis of longitudinal studies found that unipolar depression is one of the most well-investigated factors for suicidal thoughts and behaviors (22). In addition, thwarted belongingness and perceived burdensomeness could bring about depression (23, 24). Thus, we assume that depression may play an important role in the relationship between interpersonal needs and suicidal ideation.

To address the knowledge gaps, this study aims to investigate the mediating role of depression in Joiner's interpersonal theory of suicide model among transgender women in Shenyang, China. We assumed that: (1) interpersonal needs are directly associated with suicidal ideation among transgender women, and (2) depression could mediate the relationship between interpersonal needs and suicidal ideation among transgender women.

## Methods

### Study Site and Participants

With the collaboration with China Love Aid, we recruited our participants in Shenyang, China from April to July 2017. China Love Aid is a non-governmental organization (NGO) with a mission to improve the physical and mental health of transgender women. The inclusion criteria were individuals who were at least 18 years old, self-identified as transgender women, living and working in Shenyang, and agreed to participate in the study after a description of the study purposes and assurance of confidentiality. Those with obvious mental illnesses, psychological illnesses, intellectual disabilities, and unable to cooperate in completing the survey questionnaire were excluded. Finally, a total of 198 transgender women were recruited in the study.

### Recruitment and Procedures

Participants were recruited by snowball sampling, which has been commonly used to engage stakeholders from hard-to-reach vulnerable communities (25, 26). Local research assistants recruited five eligible transgender women as the “seeds.” The selected participants then recruited other eligible transgender women until the saturation met (the recruited participants could no longer introduce new participants).

Upon obtaining the informed consent among participants, research assistants provided brief instructions on how to answer the questionnaire. Then, the participants were invited to complete a paper-based questionnaire in a private room. The interviewers would assist with the request. It took an average of 30–40 min to finish the questionnaire. Upon completion, each participant received an incentive equivalent to 30 U.S. dollars as a token of appreciation for their participation. The study protocol was approved by the Institutional Review Boards at the School of Public Health of Shanghai Jiao Tong University, China (Protocol # 2016022).

## Measures

### Sociodemographic Characteristics

Demographic characteristics included age group by quartiles (1 = 18–27, 2 = 28–38, and 3 = 39–62), levels of education (1 ≤ Primary school, 2 = Junior school, 3 = High school, and 4 ≥ College school), current marital status (1 = Never married, 2 = Married with a female, and 3 = Divorced or widowed), monthly income in Chinese currency Yuan (CYN; one Yuan equals to about 0.1428 USD at the time of the survey) (1 ≤ 3,150 CYN, 2 = 3,150–6,300 CYN, 3 = 6,300–12,600 CYN, and 4 ≥ 12,600 CYN), and duration of staying in Shenyang (1 = Local, 2 = Not a local and lived for <1 year, 3 = Not a local resident and lived for 1–5 years, and 4 = Not a local and lived for over 5 years). Participants were also asked to report their sexual orientation (1 = Heterosexual, 2 = Homosexual, and 3 = Bisexual/ others), whether have received HIV-related education (1 = Yes and 0 = No), and ever made feminization changes (1 = Yes and 0 = No). HIV status was recorded based on self-report (1 = Positive, 2 = Negative, and 3 = Unknown).

### Suicidal Ideation

Participants were asked whether they had ever considered suicide in their lifetimes. Participants who answered “yes” were considered as having suicidal ideation. Several studies have used this method for suicidal ideation measurement (27–31).

### Interpersonal Needs

Both thwarted belongingness and perceived burdensomeness were measured using the Interpersonal Needs Questionnaire-15 (INQ-15) derived from the interpersonal theory of suicide (32). The INQ-15 (33, 34) is a self-report measurement with response options ranging from 1 (not at all true for me) to 7 (very true). On this scale, there were 9-item for assessing thwarted belongingness and 6 items for perceived burdensomeness. Item sample of thwarted belongingness is “These days I feel like I belong” (reverse scored) and that of perceived burdensomeness item is “These days I think I am a burden on society” (32). With appropriate recoding, sum scores were calculated to reflect thwarted belongingness and perceived burdensome, with higher scores indicating higher thwarted belongingness and perceived burdensomeness. Both measures of thwarted belongingness and perceived burdensomeness had good reliabilities (thwarted belongingness: Cronbach's α = 0.81; perceived burdensomeness: Cronbach's α = 0.84) in the current study.

### Depression

Depression was assessed using the 9-item Patient Health Questionnaire (PHQ-9). PHQ-9 assesses affective, cognitive, behavioral, and somatic symptoms of depression in the past 2 weeks (e.g., “Little interest or pleasure in doing things”). Response options are rated from 0 (not at all) to 3 (almost every day) (35–37). The sum score (ranging from 0 to 27) was used to reflect levels of depression, with a higher score indicating more depressive symptoms. A cutoff of 10 was used to define the depressive symptoms (38, 39). The reliability of the scale for the current study was acceptable (Cronbach's α = 0.90). The continuous score of depression was used for the path model.

### Statistical Analysis

Descriptive statistics was conducted to describe sociodemographic characteristics, depression, interpersonal needs, and suicidal ideation. The chi-squared analysis and *t*-test were used to examine the association between sociodemographic characteristics, interpersonal needs, depression, and suicidal ideation.

Path analysis was employed to test the mediating role of depression in the relationship between interpersonal needs and suicidal ideation after adjusting for potential confounders. The direct and indirect effects were examined by a bias-corrected bootstrap procedure based on 1,000 bootstrap samples (40). As suicidal ideation is a binary variable, the parameters were estimated using the robust weighted least squares (WLS) approach (estimator = WLSMV in Mplus). Bias-corrected confidence intervals for the direct and indirect paths were reported.

Multiple indices were used to evaluate model fits. These indices included χ^2^/*df*, comparative fit index (CFI), root mean square error of approximation (RMSEA), standardized root mean square residual (SRMR), and weighted root mean square residual (WRMR). χ^2^/*df* < 3, CFI > 0.95, RMSEA ≤ 0.06, SRMR ≤ 0.08, and WRMR ≤ 1.00 indicated better model fit (41).

Descriptive statistics and bivariate analyses were performed using the SPSS Statistics (version 23.0 for Windows9, IBM, Armonk, NY, USA). Path analysis was performed using Mplus version 7.0.

## Results

### Descriptive Statistics

The results of the descriptive analysis are demonstrated in Table 1. The majority (77.27%, *n* = 153) of participants were unmarried. There were nearly 50% of the participants aged between 28 and 38 years. Nearly 70% of the participants were homosexual. All the participants have ever made feminization changes. More than half of the participants had received HIV-related education (65.66%). About 13.13% of the participants reported an unknown HIV status and 24.75% had been diagnosed as HIV-positive.

**Table 1:** Background characteristics of transgender women (*N* = 198).

**Variables**	**Number of participants**	**Have suicidal ideation**			
	***N* (row%)**	**Yes [*n* (row%)/ Mean ± SD]**	**No [*n* (row%)/ Mean ± SD]**	**χ^2^/t**	***p***
Total	198 (100)	73 (36.87)	125 (63.13)		
Age (y)				2.44	0.295
18–27	57 (28.78)	25 (43.86)	32 (56.14)		
28–38	95 (47.98)	30 (31.58)	65 (68.42)		
39–62	46 (23.24)	18 (39.13)	28 (60.87)		
Education level				0.79	0.852
< Primary school	15 (7.58)	6 (40.00)	9 (60.00)		
Junior school	83 (41.92)	31 (37.35)	52 (62.65)		
High school	50 (25.25)	16 (32.00)	34 (68.00)		
>College school	50 (25.25)	20 (40.00)	30 (60.00)		
Marital status				0.01^*^	0.012
Unmarried	153 (77.27)	49 (32.02)	104 (67.97)		
Married with a female	11 (5.56)	5 (45.45)	6 (54.55)		
Divorced or widowed	34 (17.17)	19 (55.88)	15 (44.12)		
Monthly income				5.89	0.117
<3,150 CYN	46 (23.23)	22 (47.83)	24 (52.17)		
3,150–6,300 CYN	96 (48.48)	30 (31.25)	66 (68.75)		
6,300–12,600 CYN	41 (20.71)	13 (31.71)	28 (68.29)		
>12,600 CYN	15 (7.58)	8 (53.33)	7 (46.67)		
Duration of stay in this city				3.11	0.374
Local	70 (35.35)	31 (44.29)	39 (55.71)		
Not local and lived for less than1 year	19 (9.60)	5 (26.32)	14 (73.68)		
Not local and lived for 1 to 5 years	50 (25.25)	16 (32.00)	34 (68.00)		
Not local and lived for over 5 years	59 (29.80)	21 (35.59)	38 (64.41)		
Sex orientation				0.79	0.851
Heterosexual	20 (10.10)	7 (35.00)	13 (65.00)		
Homosexual	138 (69.70)	49 (35.51)	89 (64.49)		
Bisexual	32 (16.16)	14 (43.75)	18 (56.25)		
Others	8 (4.04)	3 (37.50)	5 (62.50)		
Ever made feminization changes				–	–
Yes	198 (100)	73 (36.87)	125 (63.13)		
No	0				
Ever received HIV education				0.36	0.550
Yes	130 (65.66)	46 (35.38)	84 (64,62)		
No	68 (34.34)	27 (39.71)	41 (60.29)		
HIV testing result				3.77	0.152
Positive	49 (24.75)	26 (53.06)	23 (46.94)		
Negative	123 (62.12)	42 (34.15)	81 (65.85)		
Unknow	26 (13.13)	5 (19.23)	21 (80.77)		
Thwarted belongingness	25.83 ± 10.44	31.03 ± 10.44	22.80 ± 9.05	−5.53^*^	0.000
Perceived burdensomeness	12.02 ± 6.89	15.23 ± 7.48	10.14 ± 5.76	−5.02^*^	0.000
Depressive symptom				35.47^*^	0.000
Depressive (≥10 score)	50 (25.25)	36 (72.00)	14 (28.00)		
Not depressive (<10 score)	148 (74.75)	37 (25.00)	111 (75.00)		

*M, mean; IQR, interquartile range; VCT, voluntary counseling and testing; PHQ-9, patient health questionnaire*.

* *p < 0.05*.

### Correlations Between Demographic, Interpersonal Needs, and Suicidal Ideation

Nearly 37% of the participants reported lifetime suicidal ideation. Only marital status was significantly associated with suicidal ideation. Suicidal ideation is significantly higher among the divorced or widowed ones than among the married or unmarried ones (55.88 vs. 45.45%, 32.02%, *p* < 0.05). Since age had been found to have a strong correlation with suicidal ideation in previous studies, it was controlled as a covariate in the multivariate analysis although it was not significant in the univariate test. Thwarted belongingness and perceived burdensomeness were significantly and positively associated with suicidal ideation.

The multivariate logistic regression showed that suicidal ideation was significantly associated with thwarted belongingness (OR_m_ = 1.068 and 95% CI = 1.030–1.108) and perceived burdensomeness (OR_m_ = 1.072 and 95% CI = 1.017–1.121) after adjusting for both age group and marital status (Table 2).

**Table 2:** Logistic regression model of the suicidal ideation based on the interpersonal theory of suicide.

**Variable**	**OR_**u**_ (95%CI)**	**AOR (95%CI)**	**OR_**m**_ (95%CI)**
Thwarted belongingness	1.089 (1.053–1.127)	1.086 (1.050–1.124)	1.068 (1.030–1.108)
Perceived burdensomeness	1.125 (1.070–1.183)	1.113 (1.058–1.172)	1.072 (1.017–1.131)

*ORu, crude odds ratio; AOR, adjusted OR, odds ratios adjusted for age group and marital status; ORm, odds ratio obtained from forward stepwise multivariate logistic regression using significant variables from the univariate analysis as input*.

### Path Model

After adjusting for marital status and age group, path model showed a good model fit (χ^2^ = 0.000, CFI = 1.000, RMSEA = 0.000, SRMR = 0.000, and WRMR = 0.17). Suicidal ideation was positively associated with thwarted belongingness and depression, and the standardized path coefficients were 0.232 (*p* = 0.021) and 0.420 (*p* < 0.01), respectively. Thwarted belongingness and perceived burdensomeness were positively associated with depression, and the standardized path coefficients were 0.462 (*p* < 0.01) and 0.222 (*p* < 0.01), respectively. The path coefficients of the final path model are shown in Table 3.

**Table 3:** Path coefficients of mediation model (*n* = 198).

**Paths**	**β**	**Std. β**	**95% CI**	**SE**	***p* value**
Thwarted belongingness → suicidal ideation	0.027	0.232	0.005–0.051	0.012	0.021
Perceived burdensomeness → suicidal ideation	0.008	0.046	−0.028–0.053	0.020	0.693
Thwarted belongingness → depression	0.121	0.462	0.050–0.191	0.034	0.000
Perceived burdensomeness → depression	0.347	0.222	0.229–0.458	0.057	0.000
Depression → suicidal ideation	0.098	0.420	0.052–0.126	0.018	0.000

*Std. β, standardized estimate; SE, standard error*.

### Mediation Analysis

**Figure 1:**
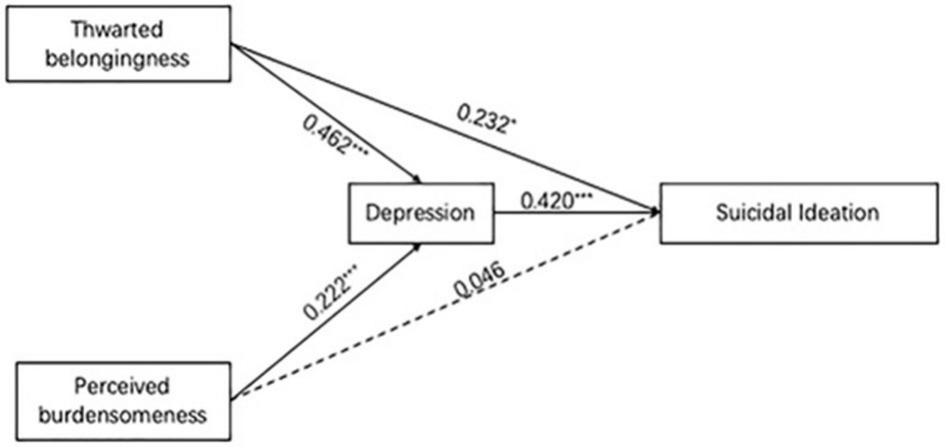
The final model of experiences of thwarted belongingness, perceived burdensomeness, depression, and suicidal ideation among transgender women (*n* = 198). All factor loadings were significant at *p* < 0.05 level. *: *p* < 0.05; ***: *p* < 0.001. Age and marital status were adjusted as covariates in the mediation model.

Results of mediation analysis were shown in Table 4 and Figure 1. The path model revealed that both the direct and indirect paths from thwarted belongingness to suicidal ideation through depression were statistically significant. There was a partial mediation effect of depression on the relationship between thwarted belongingness and suicidal ideation (indirect effect = 0.103; *p* = 0.007). The indirect path from perceived burdensomeness to suicidal ideation was statistically significant while the direct path between was not significant. There was a full mediation effect of depression on the relationship between perceived burdensomeness and suicidal ideation (indirect effect = 0.194; *p* = 0.000).

**Table 4:** Result of mediation analysis for suicidal ideation, adjusting for the effect of marital status and age group (*n* = 198).

**Effects**	**Estimate**	**Std. estimate**	**95%CI**	**SE**	***p* value**
Path 1 Thwarted belongingness → Depression → Suicidal ideation					
Total effect	0.039	0.334	0.016–0.062	0.011	0.001
Indirect effect	0.012	0.103	0.004–0.021	0.004	0.007
Direct effect	0.027	0.231	0.005–0.051	0.012	0.021
Path 2 Perceived burdensomeness → Depression → Suicidal ideation					
Total effect	0.042	0.240	0.012–0.083	0.019	0.024
Indirect effect	0.034	0.194	0.018–0.050	0.009	0.000
Direct effect	0.008	0.046	−0.028–0.053	0.020	0.693

*Total effect, = indirect effect + direct effect; Std. β, standardized estimate; SE, standard error*.

## Discussion

Transgender women suffer from elevated suicidal ideation. The study tested the interpersonal theory of suicide and examined the mediating effect of depression between interpersonal needs and suicidal ideation among transgender women in Shenyang, China. Depression fully mediated the path between perceived burdensomeness and suicidal ideation, and only showed a partial mediating effect between thwarted belongingness and suicidal ideation.

Transgender women suffered from elevated suicidal ideation. The prevalence of suicidal ideation was much higher than the general population (3.1%) in China (8). The result was consistent with the previous research (17). “Transgender” used to be regarded as a kind of mental disease until 2018 when this diagnosis was abolished by the World Health Organization. Due to the late legal identification and protection by law, transgender women are also at risk of stigmatization in China. Therefore, transgender women are marginalized in society and suffer from discrimination, including sexual abuse, verbal abuse, and are more likely to have thwarted belongingness and perceived burdensomeness than other sexual minorities populations, which may result in suicidal ideation.

Depression mediates the relationship among thwarted belongingness, perceived burdensomeness, and suicidal ideation. This finding was aligned with that of previous studies. A study by Pate and Anestis found that perceived burdensomeness and thwarted belongingness were correlated to suicidal ideation among heterosexual and sexual minorities in Mississippi, and suggested the associations between perceived burdensomeness and depression (42). People with perceived burdensomeness are more likely to have depression, which increases their risk of suicidal ideation. Thwarted belongingness is highly related to loneliness, which is also a risk factor for suicidal ideation. For thwarted belongingness, it may cause suicidal ideation through depression and other mental disorders, such as loneliness. The results of the current study suggest that transgender women experiencing increased symptoms of perceived burdensomeness and thwarted belongingness may be more likely to endorse higher levels of depression, which in turn may lead to suicidal ideation. The possible explanation for why depression incompletely mediates the relationship between thwarted belongingness and suicidal ideation while completely mediating the relationship between perceived burdensomeness and suicidal ideation is that perceived burdensomeness may not be as independently predictive of suicidal ideation as is thwarted belongingness. It may be that thwarted belongingness is most predictive of suicidal ideation when it is in the presence of perceived burdensomeness.

Unmarried transgender women were at lower risk of suicidal ideation than those who were married, divorced, or widowed. In China, transgender women are required by law to get married based on their birth sex/the sex that is documented on their birth certificates. The impact of cultural and societal stressors due to separation and divorced and financial problems may contribute to the mental disorders and suicidal ideation among them. It could also be a traumatic and stigma event for transgender women (43, 44). Also, the small sample size of this population may limit the statistical power to detect significant relationships. Future studies with a large sample size are needed to confirm our findings.

This research provides a theoretical basis for future prevention and control of suicide behaviors among transgender women. To our knowledge, this is the first study of transgender women with the interpersonal theory of suicide in China. The finding from this study could explain the causes of suicidal ideation among transgender women and provide empirical evidence for future interventions. However, there are some limitations needed to acknowledge. First, a study design of cross-sectional limits our ability to confirm the causal relationships among variables of interest. Longitudinal studies are needed to further examine our research hypotheses. Second, self-report measures were employed in this study, and self-report bias might exist. For instance, due to social desirability bias, the rate of suicidal ideation might be underestimated. Third, transgender women sampling is a very difficult process and with potential for selection bias. In this study, selection bias may lead to findings that are biased not only in the description of important data (e.g., the prevalence of suicide) but also in measures of association. Besides, suicidal ideation was assessed using one question. Although the measurement scale has been proved in previous studies, it might not have strong reliability and threaten the internal validity of our findings. Finally, the study samples were recruited from one NGO, which might limit the generalizability of our findings to other areas of China or other countries.

## Conclusion

The interpersonal theory of suicide and the mediation model could be applied to transgender women and explain the elevated suicidal ideation among them. Both perceived burdensomeness and thwarted belongingness are the facilitators of suicidal ideation, and depression could mediate between them. To reduce suicidal ideation in this population, interventions targeting both interpersonal needs and depression are needed.

## Data Availability Statement

The original contributions presented in the study are included in the article/supplementary material, further inquiries can be directed to the corresponding author/s.

## Ethics Statement

The studies involving human participants were reviewed and approved by the Institutional Review Boards at the School of Public Health of Shanghai Jiao Tong University, China (Protocol # 2016022). Written informed consent was obtained from the participants of the study. The patients/participants provided their written informed consent to participate in this study.

## Author Contributions

YC, YW, and RC designed the study. RC and CZ selected and processed the data and wrote the manuscript. XL, SQ, and HW edited and revised the manuscript. CX, XY, and TM provided useful information. All authors contributed to the subsequent drafts, reviewed, and endorsed the final submission.

## Funding

This work was supported by the National Natural Science Foundation of China [Grant Numbers 71603166 and 71673187] and Shanghai Three-year Action Plan for Public Health under Grant [GWV-10.1-XK15, GWV-10.2-XD13, and GWV-10.1-XK18].

## Conflict of Interest

The authors declare that the research was conducted in the absence of any commercial or financial relationships that could be construed as a potential conflict of interest.

## Publisher's Note

All claims expressed in this article are solely those of the authors and do not necessarily represent those of their affiliated organizations, or those of the publisher, the editors and the reviewers. Any product that may be evaluated in this article, or claim that may be made by its manufacturer, is not guaranteed or endorsed by the publisher.
